# Expression of neural cell adhesion molecule and polysialic acid in human bone marrow-derived mesenchymal stromal cells

**DOI:** 10.1186/s13287-016-0373-5

**Published:** 2016-08-15

**Authors:** Maria S. Skog, Johanna Nystedt, Matti Korhonen, Heidi Anderson, Timo A. Lehti, Maria I. Pajunen, Jukka Finne

**Affiliations:** 1 Biochemistry and Biotechnology, Department of Biosciences, University of Helsinki, P.O. Box 56, FI-00014 Helsinki, Finland; 2 Cell Therapy Services, Finnish Red Cross Blood Service, Kivihaantie 7, FI-00310 Helsinki, Finland; 3 Present Address: Genoscoper Laboratories Oy, P.O. Box 1040, FI-00251 Helsinki, Finland; 4 Present Address: Department of Bacteriology and Immunology, Medicum, Research Programs Unit, Immunobiology, University of Helsinki, P.O. Box 21, FI-00014 Helsinki, Finland

**Keywords:** Bone marrow, Mesenchymal stromal cell, Clinical grade, Neural cell adhesion molecule, Polysialic acid

## Abstract

**Background:**

In order to develop novel clinical applications and to gain insights into possible therapeutic mechanisms, detailed molecular characterization of human bone marrow-derived mesenchymal stromal cells (hBM-MSCs) is needed. Neural cell adhesion molecule (NCAM, CD56) is a transmembrane glycoprotein modulating cell–cell and cell–matrix interactions. An additional post-translational modification of NCAM is the α2,8-linked polysialic acid (polySia). Because of its background, NCAM is often considered a marker of neural lineage commitment. Generally, hBM-MSCs are considered to be devoid of NCAM expression, but more rigorous characterization is needed.

**Methods:**

We have studied NCAM and polySia expression in five hBM-MSC lines at mRNA and protein levels. Cell surface localization was confirmed by immunofluorescence staining and expression frequency in the donor-specific lines by flow cytometry. For the detection of poorly immunogenic polySia, a fluorochrome-tagged catalytically defective enzyme was employed.

**Results:**

All five known NCAM isoforms are expressed in these cells at mRNA level and the three main isoforms are present at protein level. Both polysialyltransferases, generally responsible for NCAM polysialylation, are expressed at mRNA level, but only very few cells express polySia at the cell surface.

**Conclusions:**

Our results underline the need for a careful control of methods and conditions in the characterization of MSCs. This study shows that, against the generally held view, clinical-grade hBM-MSCs do express NCAM. In contrast, although both polysialyltransferase genes are transcribed in these cells, very few express polySia at the cell surface. NCAM and polySia represent new candidate molecules for influencing MSC interactions.

## Background

Human bone marrow-derived mesenchymal stromal cells (hBM-MSCs) are attractive candidates for cellular therapy, regenerative medicine, and tissue engineering. They are adult progenitor cells that hold potential for fast clonal expansion, secretion of trophic and immunomodulatory factors, and multilineage differentiation. In addition, they are relatively easy to harvest, isolate, and culture. However, it is widely acknowledged that following in-vitro culture, MSCs undergo replicative senescence and appear to lose their beneficial traits [1]. Furthermore, MSC cultures typically consist of a heterogeneous mixture of cells at different stages of commitment and potential [1, 2]. In order to develop novel clinical applications and to gain more insight into the possible therapeutic mechanisms, detailed molecular characterization of hBM-MSCs is needed. This will ultimately improve the safety and efficacy of cell therapy.

Neural cell adhesion molecule (NCAM, CD56) is a calcium-independent binding protein engaged in homophilic cell–cell and heterophilic cell–matrix interactions [3]. NCAM is transcribed from a single gene, but exists in multiple isoforms as a result of alternative splicing [4]. In humans, at least five distinct NCAM isoforms are known. A modification of NCAM results post-translationally from the addition of linear polymers of *N*-acetylneuraminic acid units, polysialic acid (polySia) [5, 6]. Being a large negatively charged and highly hydrated structure, polySia regulates NCAM activity by altering its biophysical properties [7]. PolySia has an important role in maintaining developmental plasticity and cell migration in tissues by influencing cellular interactions [8]. In addition, it is involved in various medical conditions such as tissue repair, neurodegenerative diseases, and progression of metastatic cancers [9, 10].

Expression of NCAM and polySia is strong and dynamic during embryogenesis, decreases and focuses during development, and is limited to just few tissues and cell types in the adult [11, 12]. Because of its original discovery site, NCAM is often considered a marker of neural lineage commitment [13, 14]. However, it is known that expression of NCAM and polySia is widespread during organogenesis, particularly in undifferentiated mesenchymal cells [15]. Many in-vivo and in-vitro studies in animal models suggest that NCAM is an important regulator of cell migration and condensation during skeletal development [16–18]. Hence, NCAM and polySia represent promising candidates for influencing MSC interactions.

It is generally held that hBM-MSCs do not express NCAM [19–25], while, for example, placental and umbilical cord blood-derived MSCs are eminently NCAM positive [21, 24, 25]. Brooke et al. [26] have reported that hBM-MSCs do express *NCAM* at the mRNA level, but protein expression was not investigated. NCAM protein expression, which may indicate increased chondrogenic potential, has been reported in a small fraction of primary bone marrow mononuclear cells (0.5–5.5 %), but expression diminished over time in culture [27, 28]. In contrast, murine BM-MSCs predominantly express NCAM, which plays a crucial role, for example, in hematopoiesis [29]. Furthermore, experiments with *NCAM* knockout mice have shown reduced multilineage differentiation potential of BM-MSCs compared with wild-type controls [30, 31]. Thus, because of the role of NCAM and polySia in the control of cellular differentiation and interaction, it is important to reliably determine whether they are expressed in clinical-grade hBM-MSCs.

In this study, we have investigated the expression status of NCAM and polySia in clinical-grade hBM-MSCs using a variety of methods. We have concentrated particularly on NCAM expression, because we observed a striking discrepancy between our findings and previous reports [19–25]. Furthermore, NCAM is the most studied molecule of the immunoglobulin superfamily of cell adhesion molecules (CAMs), but has been largely neglected in stem cell research despite its role as a developmental regulator. This study clearly demonstrates the need for comprehensive analyses and careful control of methods in the characterization of MSCs. Gene and protein expression analyses show that these cells do, in fact, express NCAM. In contrast, although polysialyltransferases are transcribed in these cells, very few express polySia on the cell surface.

## Methods

### Cells

The culture protocol developed by Laitinen et al. [32] for clinical-grade MSCs based on platelet lysate was utilized in this study. Bone marrow was collected from five healthy volunteer donors (donor 067: female, age 24; donor 068: female, age 31; donor 069: female, age 30; donor 072: female, age 21; donor 073: female, age 21). Bone marrow was aspirated under local anesthesia from the posterior iliac crest and collected in heparinized tubes after signed informed consent according to the Declaration of Helsinki. The protocol was approved by the ethics committee of the Hospital District of Helsinki and Uusimaa (Finland). The isolation and characterization of hBM-MSCs has been described in detail previously [32]. The isolated cells were cultured in heparinized (LEO Pharma, Ballerup, Denmark) low-glucose Dulbecco’s modified Eagle’s medium (DMEM; Gibco, Life Technologies, Paisley, UK), supplemented with 10 % platelet lysate (Finnish Red Cross Blood Service, Helsinki, Finland), and 100 U/ml penicillin and 100 μg/ml streptomycin (Gibco) according to Laitinen et al. [32]. The medium was changed twice weekly and the cultures were passaged when subconfluent (80 % confluency) and subcultured at 1000–1500 cells/cm^2^. The hBM-MSCs used in this study were freshly analyzed (i.e., noncryopreserved) at passage 2 or 3.

Human neuroblastoma SK-N-SH cells (ATCC, Manassas, VA, USA) were cultured in high-glucose DMEM (Sigma, St. Louis, MO, USA), supplemented with 10 % fetal bovine serum (FBS) (HyClone; Thermo Scientific, Logan, UT, USA), and 100 U/ml penicillin and 100 μg/ml streptomycin (Gibco). Inactive endosialidase-GFP fusion protein developed by Jokilammi et al. [33] and magnetic GFP-Trap®-M beads (Chromotek, Planegg-Martinsried, Germany) were employed to fractionate the strongly polySia-expressing cell population (kSK-N-SH) to be used as a positive control. First, cells were labeled with inactive endosialidase-GFP fusion protein in phosphate-buffered saline (PBS; containing 1.06 mM potassium phosphate monobasic, 155.2 mM sodium chloride, and 2.97 mM sodium phosphate dibasic) for 1 hour on ice. Labeled cells were then mixed with GFP-Trap®-M beads and separated magnetically until the bead-associated cells were perceptibly gathered to the proximity of the magnet. Supernatant was discarded and isolated cells were washed with PBS. Washing and magnetic separation was repeated 10 times. Lastly, the isolated cells were plated on a cell culture dish and cultured accordingly. The NCAM and polySia expression status was analyzed with flow cytometry; the proportion of NCAM and polySia-expressing cells was 98.3 %.

All cells were cultured under a humidified atmosphere at 37 °C and with 5 % CO_2_.

### Differentiation of hBM-MSCs

Adipogenic differentiation of hBM-MSCs was performed as described previously by Laitinen et al. [32]. The cells were cultured in adipogenic differentiation condition for 1–2 weeks. After differentiation the cells were fixed with 4 % paraformaldehyde in PBS for Sudan III staining.

Osteogenic differentiation of hBM-MSCs was carried out essentially as described previously by Laitinen et al. [32]. The cells were cultured in osteogenic medium for 3–4 weeks. After differentiation the cells were fixed with 4 % paraformaldehyde in PBS for von Kossa staining.

Chondrogenic differentiation was performed as described previously by Skog et al. [34]. The pellet cultures were maintained up to 4 weeks, changing the medium twice a week. After differentiation the cell pellets were fixed with 10 % formalin, embedded in paraffin, and cut into 5 μm sections for Safranin O staining.

### Qualitative reverse transcription PCR

*NCAM* isoform-specific sequences were obtained from NCBI Unigene (http://www.ncbi.nlm.nih.gov/unigene). Primers were designed using NCBI Primer Blast (http://www.ncbi.nlm.nih.gov/tools/primer-blast) and optimized by OligoEvaluator™ (http://www.sigmaaldrich.com/technical-documents/articles/biology/oligo-evaluator.html) or Tm Calculator (https://www.thermofisher.com/fi/en/home/brands/thermo-scientific/molecular-biology/molecular-biology-learning-center/molecular-biology-resource-library/thermo-scientific-web-tools/tm-calculator.html) PCR web tools. All commercial kits were used according to the manufacturer’s instructions. Total RNA was isolated using the High Pure RNA Isolation Kit (Roche Diagnostics, Mannheim, Germany). Reverse transcription was performed with High Capacity RNA-to-cDNA Kit (Applied Biosystems, Foster City, CA, USA) with 1 μg of total RNA. A control reaction omitting the reverse transcriptase was prepared for each sample. PCRs were performed in 20 μl final volume with Phusion Hot Start II High-Fidelity DNA Polymerase (Thermo Scientific, Waltham, MA, USA) for all *NCAM* isoforms, polysialyltransferases (*ST8SIA2* and *ST8SIA4*), and controls, except that OneTaq Hot Start DNA Polymerase (New England BioLabs, Ipswich, MA, USA) was used for isoform *NCAM-120*.

Human *NCAM* isoform and polysialyltransferase-specific PCR primers were used for the amplification (Table 1). For Phusion Hot Start II High-Fidelity DNA Polymerase, the reaction mixture was composed of 0.02 U/μl of DNA polymerase, Phusion HF Buffer (with 1.5 mM MgCl_2_), 0.2 mM dNTPs, 0.5 μM primers, 3 % DMSO, sterile water (Baxter Healthcare, Zürich, Switzerland), and 2.0 μl of template. For OneTaq Hot Start DNA Polymerase, the reaction mixture was composed of 0.025 U/μl of DNA polymerase, OneTaq Standard Reaction Buffer (with 1.8 mM MgCl_2_), 0.2 mM dNTPs, 0.2 μM primers, 1.5 M betaine (Sigma), sterile water (Baxter Healthcare), and 2 μl of template. Amplification was performed for 35 cycles. The PCR products were analyzed by agarose gel electrophoresis. Glyceraldehyde 3-phosphate dehydrogenase (*GAPDH*) was used as the reference gene.

**Table 1:** Primers used in the study

Primer	UniGene	Forward (5′➔3′)	Reverse (5′➔3′)	Amplicon size (base pairs)
GAPDH	Hs.544577	GAAGGTGAAGGTCGGAGTC	GAAGATGGTGATGGGATTTC	225
NCAM-All	Hs.503878	GGACTTCTACCCGGAACATCAG	CGAGCTTAGGTGCACTGGG	
NCAM-140 (Variant 1), NCAM-125 (Variant 4)				798
NCAM-180 (Variant 2)				828
NCAM-120 (Variant 3)				903
NCAM Variant 5				906
NCAM-180	NM_181351.4	GCTTCGTGGACTCGACCAG	GCAGATGTACTCTCCGGCATC	126
NCAM-140	NM_000615.6	CGAAGAAAAGACTCTGGATGG	TCATGCTTTGCTCTCGTTCT	1498
NCAM-125/140	NM_001242608.1 / NM_000615.6	CGAAGAAAAGACTCTGGATGG	GTTCCCCTTGGACTGGC	755
NCAM-125	NM_001242608.1	CAGCCAGTCCAAGGGG	TGTAGGATGCAGAATTGCCTC	346
NCAM120/125	NM_001076682.3 / NM_001242608.1	GAGTATGAGGTCTACGTGGTGGC	GCAGAGAAAAGCAATGAGACAAAG	152
NCAM-120	NM_001076682.3	TCTGCTAGCTCGTCTACCCC	CCAAAGGGGGCACTGATCTT	541
NCAM Variant 5	NM_001242607.1	GCTTCGTGGACTCGACCAG	GCAGATGTACTCTCCGGCATC	204
ST8SIA2	Hs.302341	GGGCAACTCGGGGGTCTTGC	AAGGCCCGCTGGATGACCGA	162
ST8SIA4	Hs.308628	ACAGGCGCCGGACACTAAACA	TGCAGCAAACTCCACCACAGGA	200

### Western blot

Whole-cell extracts were prepared by lysing the cells in radioimmunoprecipitation assay (RIPA) buffer pH 6.8 containing 50 mM Trizma® base, 150 mM sodium chloride, 0.5 % sodium deoxycholate (all from Sigma), and 1 % Triton X-100 (Roche Life Sciences, Indianapolis, IN, USA), supplemented with EDTA-free protease inhibitor cocktail (Thermo Scientific, Rockford, IL, USA). For removal of polySia, 10 μg/ml of active endosialidase-GFP fusion protein [35] was added and the samples were incubated at 37 °C for 45 minutes. Samples were mixed with Laemmli sample buffer, loaded, and run on 4–20 % Mini-PROTEAN® TGX™ Precast gradient gel (Bio-Rad Laboratories, Hercules, CA, USA) or 7 % SDS-polyacrylamide gel. Proteins were electrotransferred from the gels to nitrocellulose membranes (GE Healthcare, Little Chalfont, UK) overnight at 4 °C. The membrane was blocked with 3 % skimmed milk powder in PBS with 0.1 % Tween-20 for 3 hours at 4 °C.

For NCAM detection, the membrane was incubated with a primary antibody mixture containing 0.33 μg/ml rabbit polyclonal anti-human NCAM antibody (AB5032; Millipore, Temecula, CA, USA) and 0.067 μg/ml mouse monoclonal anti-human NCAM antibody (123C3; Santa Cruz, Dallas, Texas, USA) in blocking solution overnight at 4 °C. Because anti-NCAM antibodies may detect different isoforms with varying affinity in western blot, two primary antibodies were used simultaneously to ensure accurate detection of all isoforms [36]. After three washes, the membrane was incubated with a secondary antibody mixture containing HRP-conjugated anti-rabbit secondary antibody 1:3000 and anti-mouse secondary antibody 1:3000 (both from Cell Signaling, Danvers, MA, USA) in blocking solution for 2 hours at room temperature. The immunoblots were developed with SuperSignal® West Pico Chemiluminescent Substrate (Thermo Scientific). Secondary antibody control confirmed the specificity of the labeling, and α-Tubulin (B-51-2; Sigma) served as a loading control.

### Flow cytometry

Cells were double labeled with 10 μg/ml of rabbit polyclonal anti-human NCAM antibody (AB5032; Millipore) followed by AlexaFluor 647-labeled goat anti-rabbit secondary antibody (Molecular Probes, Invitrogen, Eugene, OR, USA) and inactive endosialidase-GFP fusion protein [33] in PBS. Parallel samples were labeled with AlexaFluor 647 mouse anti-human alkaline phosphatase antibody (B4-78; BD Biosciences, San Jose, CA, USA) and FITC mouse anti-human CD44 antibody (BD Biosciences) according to the manufacturer’s instructions. Appropriate fluorescence minus one (FMO) controls were used for analysis. The cells were analyzed with FACS LSR II flow cytometer and FACSDiva 5.0.3 software (BD Biosciences). Cell debris and dead cells were excluded from the analysis based on physical parameters and propidium iodide (PI) fluorescence probing for cell viability (proportion of positive cells 1.2–7.0 %, data not shown).

### Immunocytochemistry

For immunocytochemical staining, cells were grown on glass coverslips and fixed with 4 % paraformaldehyde in PBS. Nonspecific binding was blocked with 1.5 % normal horse serum (Vector Laboratories, Burlingame, CA, USA) in PBS. Cells were labeled with 10 μg/ml of rabbit polyclonal anti-human NCAM antibody (AB5032; Millipore) followed by AlexaFluor 647 conjugated goat anti-rabbit secondary antibody (Molecular Probes) and inactive endosialidase-GFP fusion protein [33], all in PBS. Cover slips were mounted with ProLong Mounting Medium with DAPI (Molecular Probes). The staining was visualized with an Olympus BX50F-3 microscope and imaged by a PCO CCD Imaging SensiCam color camera and Image-Pro Plus 4.0 software.

## Results

### Multilineage differentiation of hBM-MSCs

**Fig. 1:**
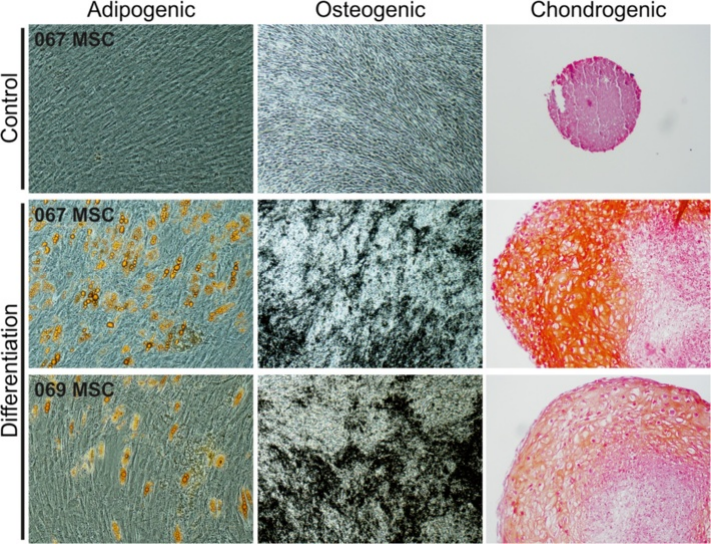
Multilineage differentiation of hBM-MSCs. Representative light microscopic photographs of control and differentiated hBM-MSCs. Adipogenic differentiation was confirmed by Sudan III staining, osteogenic differentiation by von Kossa staining, and chondrogenic differentiation by Safranin O staining. All hBM-MSC lines displayed typical differentiation capacity. Adipogenic and chondrogenic differentiation images were taken at 10× lens objective magnification, osteogenic differentiation images at 4× lens objective magnification. *MSC* mesenchymal stromal cell

The multilineage differentiation assay was performed to verify the differentiation capacity of hBM-MSCs according to the International Society for Cellular Therapy (ISCT) minimal criteria for MSCs [37]. All hBM-MSC lines displayed typical MSC differentiation capacity along the adipogenic, osteogenic, and chondrogenic lineages (Fig. 1).

### Expression of NCAM and polysialyltransferase mRNA in hBM-MSCs

**Fig. 2:**
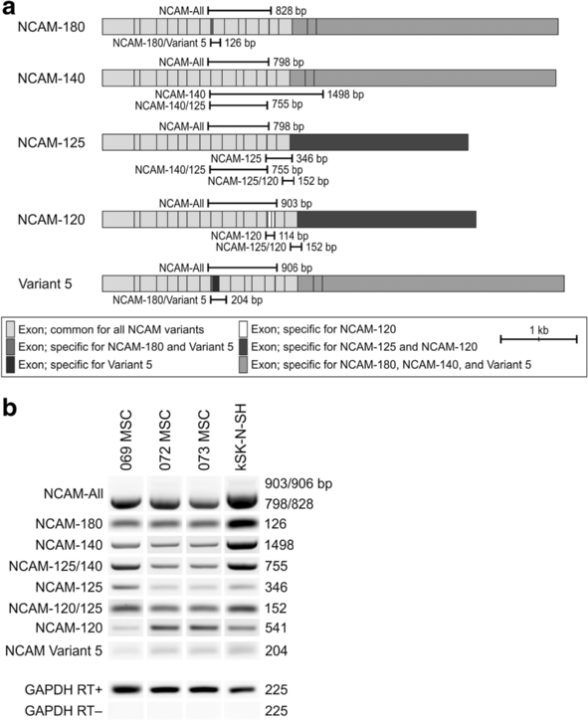
*NCAM* transcription in hBM-MSCs. NCAM is transcribed from a single gene, located in chromosome 11q23.1, consisting of at least 25 exons. As a result of alternative splicing, the protein exists in multiple isoforms. Qualitative reverse transcription PCR analysis was performed to gain an overview of *NCAM* mRNA expression. **a** Five distinct isoforms are known and can be detected with a common primer pair (NCAM-All) or isoform-specific primers as indicated. **b** All known *NCAM* isoforms are expressed in hBM-MSCs at the mRNA level. *NCAM*-expressing neuroblastoma cell line kSK-N-SH served as a control. *bp* base pairs, *GAPDH* glyceraldehyde 3-phosphate dehydrogenase, *MSC* mesenchymal stromal cell, *NCAM* neural cell adhesion molecule, *RT* reverse transcriptase

To gain an overview of *NCAM* mRNA expression, qualitative reverse transcription PCR analysis was performed with isoform-specific primer pairs (Fig. 2a). The reverse transcription PCR analysis revealed distinct expression of all NCAM isoform mRNAs in the three analyzed hBM-MSC lines (Fig. 2b).

**Fig. 3:**
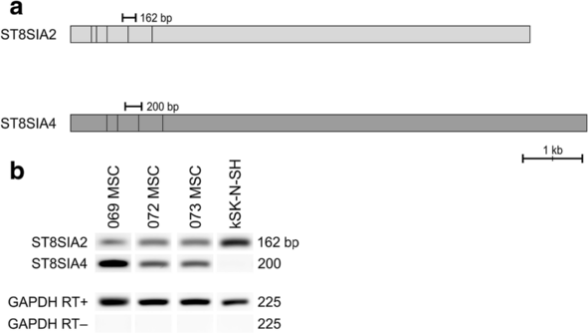
Polysialyltransferase transcription in hBM-MSCs. NCAM polysialylation is catalyzed by two Golgi resident enzymes, polysialyltransferases ST8SIA2 and ST8SIA4. Qualitative reverse transcription PCR analysis was performed to indicate the presence of polysialyltransferases. **a** *ST8SIA2* gene is located in chromosome 15q26 and consists of six exons, whereas *ST8SIA4* gene is located in chromosome 5q21 and is comprised of five exons. **b** Both polysialyltransferases are transcribed in hBM-MSCs. The polySia-expressing neuroblastoma cell line kSK-N-SH was used as a control. *bp* base pairs, *GAPDH* glyceraldehyde 3-phosphate dehydrogenase, *MSC* mesenchymal stromal cell, *RT *reverse transcriptase

NCAM polysialylation is catalyzed by two Golgi resident enzymes, polysialyltransferases ST8SIA2 and ST8SIA4 (Fig. 3a). Transcription of polysialyltransferases was analyzed to confirm the prospect of NCAM polysialylation. The analysis shows unambiguously that both transferase mRNAs are transcribed in the three hBM-MSC lines analyzed (Fig. 3b).

### Surface expression of NCAM and polySia in hBM-MSCs

**Fig. 4:**
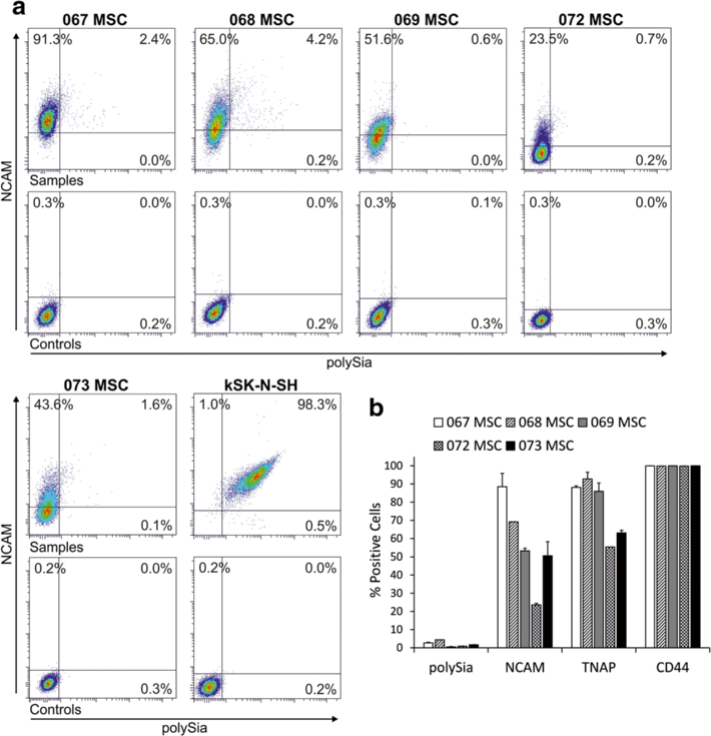
Flow cytometric analysis of marker expression in hBM-MSCs. Flow cytometric analyses were performed to determine the prevalence of marker expression in hBM-MSC lines. **a** Plots of NCAM antibody and endosialidase-GFP fusion protein-labeled cells shown in comparison with plots of secondary antibody controls. NCAM was detected in all hBM-MSC lines, but the proportion of positive cells varied. In contrast, the proportion of polySia positive cells was very low. The NCAM and polySia-expressing cell line kSK-N-SH served as a control. **b** For comparison, hBM-MSC lines were analyzed for the expression of CD44, a common MSC marker, and tissue nonspecific alkaline phosphatase (*TNAP*), an early osteogenic marker. Standard deviation is indicated. No correlation between marker expressions was detected. *MSC* mesenchymal stromal cell, *NCAM* neural cell adhesion molecule, *polySia* polysialic acid

Using commercially available polyclonal human-specific NCAM antibody, we detected cell surface NCAM expression on hBM-MSCs in flow cytometry (Fig. 4a), indicating that *NCAM* mRNA is translated into protein. All five hBM-MSC lines included in this study expressed NCAM on the cell surface. However, its occurrence was heterogeneous, because not all cells in the populations expressed NCAM at detectable levels. Differences between donor-specific hBM-MSC lines were also observed, the proportion of positive cells ranging from 23.6 ± 0.8 % to 88.5 ± 7.4 % (Fig. 4b). Concurrent polySia detection was performed with inactive endosialidase-GFP fusion protein that binds to polySia. Surprisingly, only very few hBM-MSCs expressed polySia (Fig. 4a), the proportion of positive cells ranging from 0.5 ± 0.2 % to 4.4 % (Fig. 4b). All polySia-expressing cells were simultaneously labeled positively for NCAM, the main carrier of polySia.

For comparison, cell surface expression of CD44 and tissue nonspecific alkaline phosphatase (TNAP) was also analyzed from hBM-MSC lines. Flow cytometry data indicate that CD44, a common MSC marker, is fully expressed in all of the lines (>99 %, Fig. 4b). On the other hand, expression of the early osteogenic marker TNAP was more heterogeneous, ranging from 55.3 % to 92.8 ± 3.7 % (Fig. 4b). No correlation was found between NCAM and TNAP expression.

### Expression of NCAM isoforms in hBM-MSCs

**Fig. 5:**
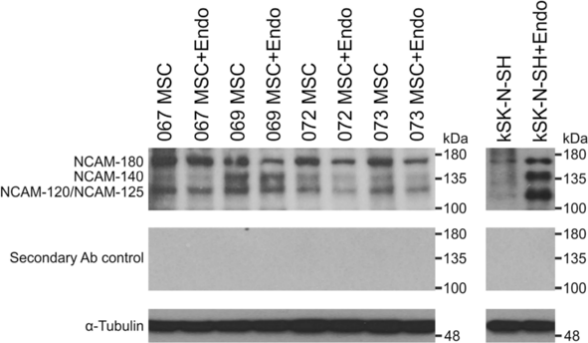
NCAM protein expression in hBM-MSCs. NCAM is expressed as transmembrane (NCAM-180 and NCAM-140), membrane-anchored (NCAM-125 and NCAM-120), or secreted (any isoform) protein. Western blotting was performed to identify which NCAM isoforms are expressed at the protein level in hBM-MSCs. In gel electrophoresis, polySia may affect the mobility of NCAM. Parallel samples were thus treated with endosialidase (*+Endo*) to remove polySia. NCAM was detected with a mixture of two primary antibodies. All of the main NCAM isoforms—NCAM-180, NCAM-140, and NCAM-120—were detected from the whole cell lysates of hBM-MSCs. The NCAM-120 and NCAM-125 isoforms could not be distinguished from one another. The NCAM-expressing neuroblastoma cell line kSK-N-SH served as a positive control. Secondary antibody control confirmed the specificity of the labeling and α-Tubulin served as a loading control. *MSC* mesenchymal stromal cell, *NCAM* neural cell adhesion molecule

NCAM protein expression was further confirmed by western blot analysis (Fig. 5). Bands of approximately 180, 140, and 120 kDa in size were detected in all hBM-MSC lines analyzed, indicating the presence of the three main isoforms of NCAM. At the protein level, hBM-MSCs seem to express predominantly NCAM-180 and other isoforms to a lesser extent. However, distinction between NCAM-125 and NCAM-120 was not possible because the bands overlap. Some variation in the expression of different isoforms was observed between the hBM-MSC lines, MSC 069 expressing different isoforms in more equal manner compared with other hBM-MSC lines. Because polySia may affect NCAM mobility in gel electrophoresis, endosialidase [35] was used to treat a set of parallel samples. In agreement with high polySia expression on kSK-N-SH cells (Fig. 5), endosialidase treatment revealed the presence of NCAM in these cells. In contrast, endosialidase treatment did not uncover additional NCAM in hBM-MSCs. Altogether, the western blot results demonstrate that hBM-MSCs express various NCAM isoforms also at the protein level.

### Cellular localization of NCAM and polySia

**Fig. 6:**
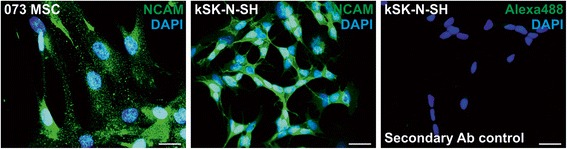
NCAM localization in hBM-MSCs. Immunofluorescence staining was performed to study NCAM localization. Exemplified fluorescence microscopic photographs of antibody-labeled nonpermeabilized cells. On hBM-MSCs, NCAM is expressed in a clustered manner around the cell surface. In contrast, NCAM expression on kSK-N-SH cells appears to be even and smooth, and concentrating towards cell–cell contact sites. Secondary antibody control confirmed the specificity of the staining. *Scale bar* = 25 μm. *DAPI* 4′,6-diamidino-2-phenylindole, *MSC* mesenchymal stromal cell, *NCAM* neural cell adhesion molecule

To determine the subcellular localization of NCAM and polySia, immunocytochemical detection was utilized. NCAM appears to be expressed in a clustered manner over the cell surface in hBM-MSCs (Fig. 6). In contrast, NCAM expression was even and smooth on the reference neuroblastoma cell line kSK-N-SH. NCAM expression in kSK-N-SH seems to be more intense around cell–cell contact sites, whereas this was not the case for hBM-MSCs.

**Fig. 7:**
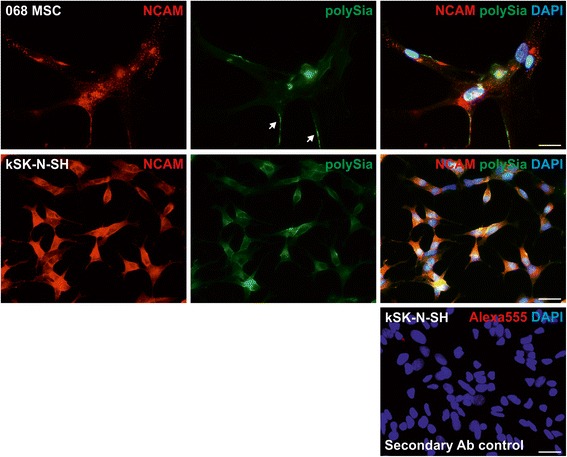
PolySia localization in hBM-MSCs. Immunofluorescence staining was performed to study polySia localization. Exemplified fluorescence microscopic photographs of antibody and fusion protein-labeled nonpermeabilized cells. Only very little polySia was detected on hBM-MSCs, mostly on cell extensions (*white arrows*). In contrast, polySia expression on kSK-N-SH cells is fairly strong and smooth throughout the cell surface. Secondary antibody control confirmed the specificity of the staining. *Scale bar* = 25 μm. *DAPI* 4′,6-diamidino-2-phenylindole, *MSC* mesenchymal stromal cell, *NCAM* neural cell adhesion molecule, *polySia* polysialic acid

Compared with NCAM, very little polySia expression was detected in hBM-MSCs (Fig. 7). Grainy polySia expression was detected mainly on cellular extensions. This was very different from the polySia expression in kSK-N-SH cells, where polySia is distributed smoothly all over the cell surface. In kSK-N-SH cells, polySia expression, together with NCAM, appears to be more intense in cell–cell contacts; however, in hBM-MSCs such condensation was not detected. Altogether, the immunocytochemistry results confirm that NCAM protein is expressed on the cell surface.

## Discussion

Previous studies have shown that NCAM is a modulator of various cellular functions such as cell–cell and cell–matrix interactions, neuronal migration, neurite outgrowth, and synapse formation [3, 38, 39]. Furthermore, it is an important regulator of cell migration and condensation during skeletal development [16–18]. It is noteworthy that NCAM is not merely an adhesion molecule, but initiates the formation of intracellular membrane-proximal signaling complexes, thereby activating a complex network of signal transduction [40]. In addition, polySia affects cellular functions such as migration, cytokine response, cell contact-dependent differentiation, and immune response modulation [41–44].

To date, definite information about NCAM and polySia expression in hBM-MSCs has been lacking. Because of the intrinsic heterogeneity of the MSC populations, donor variation, and diversity in culture conditions and analysis methods, biomolecular and cytometric characterization of MSCs from different laboratories is not easy to compare [2]. Furthermore, transcriptomic profiling has become a popular method for characterizing MSCs, but protein and mRNA expression levels do not always correlate. Analysis at the protein level is therefore needed before conclusions about the MSC phenotype can be drawn.

To gain more insight into the characteristics of xenofree hBM-MSCs, we have utilized an established clinical-grade culture protocol based on human platelet lysate [32]. Platelet lysate has been approved as a safe and effective supplement for MSC cultivation in vitro [45]. Conventional cell culture methods involve many animal-derived components; however, they are not desirable for clinical-grade cell production because of the increased risk of cross-contamination and host immune reactions [46]. In addition, xenogeneic additives, like bovine sera, may negatively alter the self-renewal and stemness of hBM-MSCs [47]. Because MSCs are very rare in the bone marrow, isolation and in-vitro expansion of the cells is usually required prior to their use. We analyzed noncryopreserved cells at passage 2 or 3, which is the time point of choice for most MSC applications because it offers the minimum required number of cells that still hold functional potency [48, 49]. NCAM and polySia expression is possibly altered during in-vitro culture. However, analysis of the properties of these cells directly from the bone marrow would be challenging because reliable markers for their identification are lacking. Culture of MSCs for at least two passages is commonly used to attain population purity [48].

Our findings regarding NCAM expression in hBM-MSCs differ from those reported previously [19–25], and show—in contrast to the generally held view—that the cells in fact do express NCAM. Furthermore, we conducted a more detailed analysis regarding NCAM gene and protein expression. Our data show that all five known *NCAM* isoforms are transcribed in hBM-MSCs. In particular, the main isoforms are detected at the protein level. NCAM protein is expressed throughout the cell surface in a clustered manner. However, flow cytometric analysis revealed quite broad donor-specific variation in expression levels between the hBM-MSC lines. This is not unexpected even in our relatively homogeneous donor population (healthy females, age 21–31 years), because it has been reported previously that hBM-MSC cultures are heterogeneous mixtures of cells, the properties and potency of which vary greatly between individual donors independent of age or gender [50–52]. In this study, the donor population was not selected based on any specific characteristic, but samples were obtained from the volunteer donors in the order they came in based on the national guidelines for bone marrow donation eligibility. In Finland, as in most other western countries, the majority of bone marrow donors are female [53]. In general, gender has little effect on hBM-MSC features [50, 54, 55].

There are many possible reasons for the conflicting reports about NCAM expression. MSCs are cultured under various conditions (e.g., FBS, human serum, platelet lysate, or serum free) and diverse procedures are employed in their management. Donor-dependent variation may also occur. Expression patterns may be regulated temporally and the cells may be in different stages and passages at the time of analysis, or have undergone replicative senescence. Also, the cellular phenotype may alter between fresh and cryopreserved cells. Technical variation may also be responsible; for example, the variable sensitivity and isoform specificity of anti-NCAM antibodies may give rise to misleadingly low or lacking protein expression. Furthermore, the expression of *NCAM* mRNA transcripts does not necessarily correlate with the expression of protein on the cell surface.

For comparison, the hBM-MSC lines were also analyzed by flow cytometry for the surface expression of CD44 and TNAP. CD44, a receptor for hyaluronic acid and a common MSC marker, is involved in the contact between stem cells and the niche for stemness maintenance, as well as MSC homing [56, 57]. All five hBM-MSC lines expressed high levels of CD44 (>99 %), as expected. TNAP is an early osteogenic marker that is expressed in a stage-specific manner during skeletal development [58]. Furthermore, TNAP deficiency causes bone hypomineralization, abnormalities in brain development, cortical malformations, as well as epileptic seizures [59]. Thus, TNAP and NCAM are developmentally involved in many of the same processes. However, no correlation between TNAP and NCAM expression was observed in the hBM-MSC lines. Also, it has been reported previously that TNAP expression may vary greatly between individual donors [50, 60] and our results further support this finding.

To our knowledge, polySia expression in hBM-MSCs has not been reported previously. Our results show that both polysialyltransferases, ST8SIA2 and ST8SIA4, catalyzing polySia synthesis are transcribed in these cells. However, very few cells express polySia on the cell surface. On closer examination it was perceived that polySia is expressed mostly on the cell extensions, in accordance with its natural role as a promoter of cell projection outgrowth and targeting [61]. Difference in polysialyltransferase expression and polysialylation levels is an interesting finding, because traditionally it is thought that expression of polySia correlates with transcription of polysialyltransferases [62, 63]. However, it has been previously shown that other, calcium-dependent, nontranscriptional regulatory pathways also exist [64]. Such nontranscriptional regulation may be due to the spatiotemporal nature of polySia, requiring specific cues for prompt and selective expression on the cell surface [65, 66].

Different cell types express different glycan signatures, a property which has also been utilized to identify and purify stem cells [67]. For example, the glycolipids SSEA-3 and SSEA-4 are amongst the most commonly used markers to identify embryonic stem cells; however, they are not necessary for the maintenance of pluripotency [68]. It is well known that expression of polysialylated NCAM decreases during postnatal development and mostly unpolysialylated NCAM is expressed in adult tissues, where it regulates cell interactions independent of polySia [69, 70]. In addition, a recent study shows that polysialylation is regulating human pluripotent stem cell differentiation into the three germ layers [63]. In the mesoderm, ST8SIA4 is the principle polysialyltransferase under normal conditions, but this switches to ST8SIA2 when ST8SIA4 activity is eliminated [63]. The observed expression pattern of polysialyltransferases and restricted polysialylation may thus indicate that hBM-MSCs are different from their prenatal pluripotent counterparts. However, our differentiation results evidently demonstrate that these cells still possess multilineage differentiation capacity. Furthermore, the uncovered polySia and NCAM expression may provide novel targets to modify MSC function [71].

## Conclusions

Despite some promising clinical results related to refractory graft-versus-host disease, the biological properties of MSCs remain largely unknown and clinical MSC applications are lacking good markers, which reflect the clinical efficacy of the cells. Generally, NCAM expression is considered to be absent from hBM-MSCs, but the results of our gene and protein expression analysis show that clinical-grade hBM-MSCs do, in fact, express NCAM as well as polySia. Hence, these results underline the need for comprehensive analyses and careful control of methods in the characterization of MSCs. NCAM and polySia represent promising new candidates to influence MSC interactions. However, their functional and clinical significance needs to be explored in further studies.

## Abbreviations

CAM, cell adhesion molecule; DAPI, 4′,6-diamidino-2-phenylindole; FBS, fetal bovine serum; FMO, fluorescence minus one; GFP, green fluorescent protein; hBM-MSC, human bone marrow-derived mesenchymal stromal cell; MSC, mesenchymal stromal cell; NCAM, neural cell adhesion molecule; PBS, phosphate-buffered saline; polySia, polysialic acid; ST8SIA2, ST8 alpha-*N*-acetyl-neuraminide alpha-2,8-sialyltransferase 2; ST8SIA4, ST8 alpha-*N*-acetyl-neuraminide alpha-2,8-sialyltransferase 4; TNAP, tissue nonspecific alkaline phosphatase
